# Dibromidochlorido{2-[(dimethyl­amino)­meth­yl]phenyl-κ^2^ *N*,*C* ^1^}tellurium(IV)

**DOI:** 10.1107/S1600536811054560

**Published:** 2011-12-23

**Authors:** Prakul Rakesh, Harkesh B. Singh, Ray J. Butcher

**Affiliations:** a Department of Chemistry, Indian Institute of Technology Bombay, Powai, Mumbai 400 076, India; b Department of Chemistry, Howard University, 525 College Street NW, Washington, DC 20059, USA

## Abstract

The title compound, C_9_H_13_Br_2_ClNTe, was synthesized by reacting [2-(dimethyl­amino­meth­yl)phen­yl]tellurium(II) chlor­ide with Br_2_. As a consequence, the Cl and Br atoms are not well ordered but distributed over the three possible positions such that the overall stiochiometry is two Br atoms and one Cl atom. The scrambling of the Br and Cl atoms indicates a small energy barrier for the exchange process between the apical and equatorial positions. Overall, the Te atom geometry is slightly distorted square pyramidal (τ = 0.052 for the major component). However, there is a weak secondary inter­action between the Te atoms and the disordered Br/Cl atoms of a nearby mol­ecule. The Te—Br and Te—Cl distances in both disorder components fall into two groups; a longer distance for the Br/Cl involved in this secondary inter­action [2.6945 (17) Å for Br and 2.601 (9)Å for Cl] and shorter bond distances to the remaining halogen atoms, indicating that this inter­action has slightly weakened the Te—*X* bond, as is the case in the previously reported tribromido structure [Singh *et al.* (1990). *J. Chem. Soc. Dalton Trans.* pp. 907–913]. Otherwise, the metrical parameters in the two structures are not significantly different. An intermolecular C—H⋯Br interaction occurs.

## Related literature

For related structures, see: Panda *et al.* (1999 ▶); Singh & McWhinnie (1985 ▶); Singh *et al.* (1992 ▶); Singh *et al.* (1990 ▶). For the synthesis of similar dibromidochlorido derivatives of tellurium, see: Rivkin *et al.* (1991 ▶); Cobbledick *et al.* (1979 ▶). For the asymmetry parameter, see: Addison *et al.* (1984 ▶). For the preparation of bis­[2-(dimethyl­amino­meth­yl)phen­yl]ditel­lur­ide, see: Kaur *et al.* (1995 ▶).
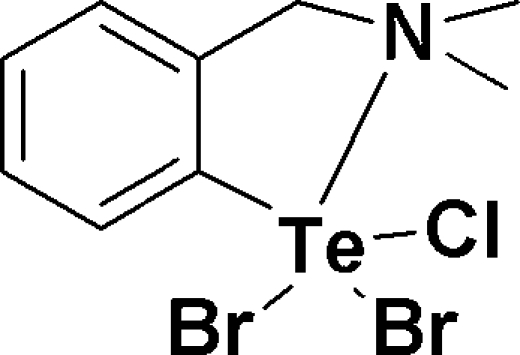


## Experimental

### 

#### Crystal data

C_9_H_12_Br_2_ClNTe
*M* *_r_* = 457.07
Monoclinic, 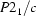

*a* = 7.2854 (3) Å
*b* = 12.4785 (5) Å
*c* = 14.4098 (6) Å
β = 98.200 (4)°
*V* = 1296.61 (9) Å^3^
*Z* = 4
Mo *K*α radiation
μ = 8.63 mm^−1^
*T* = 123 K
0.63 × 0.50 × 0.10 mm

#### Data collection

Oxford Diffraction Xcalibur Ruby Gemini diffractometer
Absorption correction: analytical [*CrysAlis PRO* (Oxford Diffraction, 2007 ▶), based on expressions derived by Clark & Reid (1995 ▶)] *T*_min_ = 0.042, *T*_max_ = 0.409
8229 measured reflections
4241 independent reflections
2981 reflections with *I* > 2σ(*I*)
*R*_int_ = 0.044

#### Refinement

*R*[*F* ^2^ > 2σ(*F* ^2^)] = 0.038
*wR*(*F* ^2^) = 0.056
*S* = 0.96
4241 reflections
141 parameters
1 restraint
H-atom parameters constrained
Δρ_max_ = 0.91 e Å^−3^
Δρ_min_ = −0.92 e Å^−3^

### 

Data collection: *CrysAlis PRO* (Oxford Diffraction, 2007 ▶); cell refinement: *CrysAlis PRO*; data reduction: *CrysAlis PRO*; program(s) used to solve structure: *SHELXS97* (Sheldrick, 2008 ▶); program(s) used to refine structure: *SHELXL97* (Sheldrick, 2008 ▶); molecular graphics: *SHELXTL* (Sheldrick, 2008 ▶); software used to prepare material for publication: *SHELXTL*.

## Supplementary Material

Crystal structure: contains datablock(s) I, global. DOI: 10.1107/S1600536811054560/jj2114sup1.cif

Structure factors: contains datablock(s) I. DOI: 10.1107/S1600536811054560/jj2114Isup2.hkl

Additional supplementary materials:  crystallographic information; 3D view; checkCIF report

## Figures and Tables

**Table 1:** Hydrogen-bond geometry (Å, °)

*D*—H⋯*A*	*D*—H	H⋯*A*	*D*⋯*A*	*D*—H⋯*A*
C7—H7*A*⋯Br2^i^	0.99	2.96	3.839 (4)	149

Symmetry code: (i) 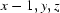.

**Fig. 1.**
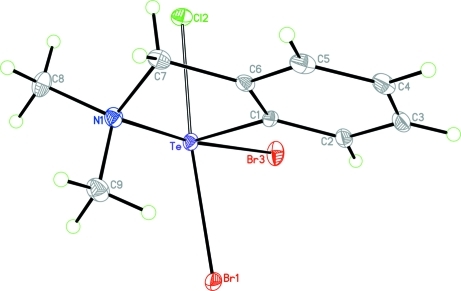
Diagram of the major component of C9H12Br2ClNTe, showing atom labeling. Atomic displacement parameters are at the 30% level.

**Fig. 2.**
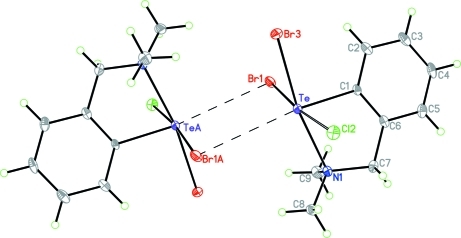
Diagram showing the formation of a dimer through weak Te···Br interactions. These interactions are shown as dashed lines

**Fig. 3.**
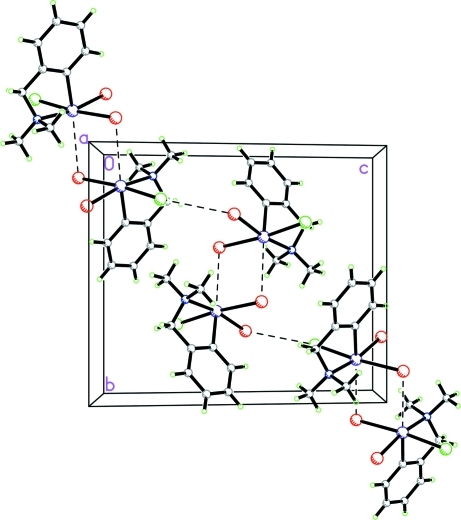
The molecular packing for C9H12Br2ClNTe viewed along the a axis. Te—Br secondary interactions are shown by dashed lines.

**Fig. 4.**
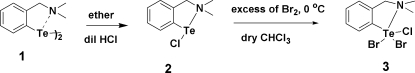
The formation of the title compound.

## supplementary crystallographic information

### Comment

Unlike their selenium analogues, simple aryltellurenyl halides are thermally unstable and polymeric in nature. However, it has been shown that they can be stabilized by intramolecular coordination and thus be isolated and structurally characterized (Singh & McWhinnie, 1985; Singh *et al.*, 1990; Singh *et al.*, 1992; Panda *et al.*, 1999). Previously the structures {2-[(*S*)-1-(dimethylamino)ethyl]phenyl}tellurium(IV) trichloride (Singh *et al.* 1992) and 2-(dimethylaminomethyl)phenyl]tellurium(IV) tribromide (Singh *et al.* 1990) have been published. In this report the structure of 2-(dimethylaminomethyl)phenyl]tellurium(IV)dibromide chloride is presented in which the 2Br's and Cl are distributed over the three possible sites. The scrambling of the Br/Cl position indicates a small energy barrier for the exchange process between the axial and equatorial positions. The synthesis of similar dibromochloro derivatives of Te have been reported previously although no crystal structures were completed (Cobbledick *et al.*, 1979; Rivkin *et al.*, 1991).

Overall the molecule is slightly distorted square pyramidal [τ = 0.052 for the major component (Addison *et al.*, 1984]. However there is a weak secondary interaction between the Te and Br/Cl of an adjoining molecule. The Te—Br and Te—Cl distances in both molecules fall into two groups; a longer distance for the Br/Cl involved in this secondary interaction (2.6945 (17)Å for Br and 2.601 (9)Å for Cl) and shorter bond distances to the remaining halogens, indicating that this interaction has slightly weakened the Te—*X* bond, as is the case in the previously reported polymorph. Otherwise, the metrical parameters in both polymorphs are not significantly different.

### Experimental

As shown in the reaction scheme (scheme 2), a stirred solution of bis[2-(dimethylaminomethyl)phenyl]ditelluride, 1, (Kaur *et al.*, 1995) (0.5 g, 0.94 mmol) in diethylether (10 ml) was treated with HCl (3 ml in 20 ml distilled water). The reaction mixture was further stirred for 10 min. The resulting reaction mixture was evaporated to one third of its original volume and ethanol (5 ml) was added to get a yellow solid. It was redissolved in ethanol and stored in the refrigerator to get yellow needles of the monochloride, 2.

A stirred solution of 2 (0.2 g, 0.66 mmol) in dry CHCl_3_ (10 ml) was treated with Br_2_ (0.37 ml, 2.34 mmol) under N_2_ at 0° C. The reaction mixture was further stirred for 2 h and then reduced to half volume and kept in freezer to give a yellow crystalline solid, 3, which contained crystals of two morphologies. This is the structure of one of these.

### Refinement

H atoms were placed in geometrically idealized positions and constrained to ride on their parent atoms with C—H distances of 0.95 - 0.97 Å [*U*_iso_(H) = 1.2*U*_eq_(OH, CH, CH_2_) [*U*_iso_(H) = 1.5*U*_eq_(CH_3_)]. As is discussed above, the 2 Br's and Cl are distributed over the three possible positions. Initially the Br/Cl occupancy in each position was refined as a free variable. These Br and Cl occupancies summed to Br_2.03_ and Cl_0.98_. The three free variables were then constrained to match a stoichiometry of Br_2_ and Cl.

### Crystal data

**Table:**

C_9_H_12_Br_2_ClNTe	*F*(000) = 848
*M**_r_* = 457.07	*D*_x_ = 2.341 Mg m^−^^3^
Monoclinic, *P*2_1_/*c*	Mo *K*α radiation, λ = 0.71073 Å
Hall symbol: -P 2ybc	Cell parameters from 3585 reflections
*a* = 7.2854 (3) Å	θ = 5.1–32.5°
*b* = 12.4785 (5) Å	µ = 8.63 mm^−^^1^
*c* = 14.4098 (6) Å	*T* = 123 K
β = 98.200 (4)°	Plate, yellow
*V* = 1296.61 (9) Å^3^	0.63 × 0.50 × 0.10 mm
*Z* = 4	

### Data collection

**Table:**

Oxford Diffraction Xcalibur Ruby Gemini diffractometer	4241 independent reflections
Radiation source: Enhance (Mo) X-ray Source	2981 reflections with *I* > 2σ(*I*)
graphite	*R*_int_ = 0.044
Detector resolution: 10.5081 pixels mm^-1^	θ_max_ = 32.6°, θ_min_ = 5.4°
ω scans	*h* = −9→11
Absorption correction: analytical [*CrysAlis PRO* (Oxford Diffraction, 2007), based on expressions derived by Clark & Reid (1995)]	*k* = −18→13
*T*_min_ = 0.042, *T*_max_ = 0.409	*l* = −16→20
8229 measured reflections	

### Refinement

**Table:**

Refinement on *F*^2^	Primary atom site location: structure-invariant direct methods
Least-squares matrix: full	Secondary atom site location: difference Fourier map
*R*[*F*^2^ > 2σ(*F*^2^)] = 0.038	Hydrogen site location: inferred from neighbouring sites
*wR*(*F*^2^) = 0.056	H-atom parameters constrained
*S* = 0.96	*w* = 1/[σ^2^(*F*_o_^2^) + (0.0084*P*)^2^] where *P* = (*F*_o_^2^ + 2*F*_c_^2^)/3
4241 reflections	(Δ/σ)_max_ = 0.001
141 parameters	Δρ_max_ = 0.91 e Å^−^^3^
1 restraint	Δρ_min_ = −0.92 e Å^−^^3^

### Special details

**Table:**

Experimental. CrysAlisPro (Oxford Diffraction, 2007) Analytical numeric absorption correction using a multifaceted crystal model based on expressions derived by R.C. Clark & J.S. Reid. (Clark, R. C. & Reid, J. S. (1995). Acta Cryst. A51, 887-897)
Geometry. All e.s.d.'s (except the e.s.d. in the dihedral angle between two l.s. planes) are estimated using the full covariance matrix. The cell e.s.d.'s are taken into account individually in the estimation of e.s.d.'s in distances, angles and torsion angles; correlations between e.s.d.'s in cell parameters are only used when they are defined by crystal symmetry. An approximate (isotropic) treatment of cell e.s.d.'s is used for estimating e.s.d.'s involving l.s. planes.
Refinement. Refinement of *F*^2^ against ALL reflections. The weighted *R*-factor *wR* and goodness of fit *S* are based on *F*^2^, conventional *R*-factors *R* are based on *F*, with *F* set to zero for negative *F*^2^. The threshold expression of *F*^2^ > σ(*F*^2^) is used only for calculating *R*-factors(gt) *etc*. and is not relevant to the choice of reflections for refinement. *R*-factors based on *F*^2^ are statistically about twice as large as those based on *F*, and *R*- factors based on ALL data will be even larger.

### Fractional atomic coordinates and isotropic or equivalent isotropic displacement parameters (Å^2^)

**Table:**

	*x*	*y*	*z*	*U*_iso_*/*U*_eq_	Occ. (<1)
Te	0.51999 (3)	0.349798 (17)	0.583822 (16)	0.01724 (6)	
Br1	0.2864 (3)	0.39052 (12)	0.42535 (11)	0.0262 (2)	0.6585 (4)
Br2	0.7403 (2)	0.28626 (15)	0.73580 (13)	0.0228 (2)	0.6374 (13)
Br3	0.7448 (2)	0.26391 (13)	0.47988 (10)	0.0291 (2)	0.7041 (13)
Cl1	0.2892 (15)	0.4029 (7)	0.4363 (6)	0.0262 (2)	0.3415 (4)
Cl2	0.7179 (11)	0.2938 (7)	0.7271 (6)	0.0228 (2)	0.3626 (13)
Cl3	0.7337 (15)	0.2589 (9)	0.4931 (7)	0.0291 (2)	0.2959 (13)
N1	0.3019 (4)	0.4005 (2)	0.6881 (2)	0.0190 (6)	
C1	0.3619 (4)	0.2094 (2)	0.5943 (2)	0.0153 (7)	
C2	0.3578 (5)	0.1257 (3)	0.5303 (3)	0.0212 (8)	
H2A	0.4292	0.1294	0.4801	0.025*	
C3	0.2486 (5)	0.0371 (3)	0.5405 (3)	0.0266 (9)	
H3A	0.2463	−0.0212	0.4980	0.032*	
C4	0.1421 (5)	0.0337 (3)	0.6134 (3)	0.0253 (9)	
H4A	0.0673	−0.0273	0.6203	0.030*	
C5	0.1438 (5)	0.1179 (3)	0.6758 (3)	0.0238 (8)	
H5A	0.0695	0.1148	0.7249	0.029*	
C6	0.2551 (4)	0.2077 (3)	0.6667 (2)	0.0180 (7)	
C7	0.2670 (5)	0.2987 (3)	0.7364 (3)	0.0210 (8)	
H7A	0.1495	0.3041	0.7632	0.025*	
H7B	0.3689	0.2852	0.7883	0.025*	
C8	0.3833 (5)	0.4816 (3)	0.7561 (3)	0.0258 (8)	
H8A	0.2979	0.4958	0.8014	0.039*	
H8B	0.4049	0.5479	0.7229	0.039*	
H8C	0.5014	0.4549	0.7892	0.039*	
C9	0.1283 (5)	0.4418 (3)	0.6349 (3)	0.0268 (9)	
H9A	0.0422	0.4613	0.6785	0.040*	
H9B	0.0719	0.3864	0.5918	0.040*	
H9C	0.1554	0.5052	0.5991	0.040*	

### Atomic displacement parameters (Å^2^)

**Table:**

	*U*^11^	*U*^22^	*U*^33^	*U*^12^	*U*^13^	*U*^23^
Te	0.02085 (12)	0.01446 (11)	0.01669 (11)	−0.00383 (10)	0.00360 (9)	−0.00064 (9)
Br1	0.0431 (3)	0.0156 (5)	0.0177 (5)	−0.0027 (3)	−0.0030 (3)	−0.0001 (3)
Br2	0.0143 (5)	0.0283 (4)	0.0240 (5)	0.0002 (3)	−0.0033 (3)	0.0020 (3)
Br3	0.0366 (4)	0.0252 (3)	0.0299 (6)	−0.0057 (3)	0.0193 (3)	−0.0062 (3)
Cl1	0.0431 (3)	0.0156 (5)	0.0177 (5)	−0.0027 (3)	−0.0030 (3)	−0.0001 (3)
Cl2	0.0143 (5)	0.0283 (4)	0.0240 (5)	0.0002 (3)	−0.0033 (3)	0.0020 (3)
Cl3	0.0366 (4)	0.0252 (3)	0.0299 (6)	−0.0057 (3)	0.0193 (3)	−0.0062 (3)
N1	0.0209 (16)	0.0156 (14)	0.0207 (16)	−0.0032 (12)	0.0037 (13)	0.0001 (11)
C1	0.0146 (17)	0.0139 (16)	0.0159 (17)	−0.0063 (14)	−0.0032 (14)	0.0001 (12)
C2	0.0176 (18)	0.0156 (18)	0.028 (2)	0.0011 (14)	−0.0056 (15)	−0.0009 (14)
C3	0.027 (2)	0.0149 (18)	0.034 (2)	0.0026 (16)	−0.0091 (17)	0.0010 (15)
C4	0.0190 (19)	0.0184 (18)	0.035 (2)	−0.0076 (16)	−0.0087 (17)	0.0080 (15)
C5	0.0184 (19)	0.025 (2)	0.026 (2)	−0.0041 (16)	−0.0018 (16)	0.0085 (15)
C6	0.0157 (18)	0.0150 (17)	0.0209 (19)	−0.0015 (14)	−0.0055 (14)	0.0028 (13)
C7	0.0185 (19)	0.0224 (19)	0.023 (2)	0.0002 (15)	0.0047 (15)	0.0055 (14)
C8	0.025 (2)	0.026 (2)	0.027 (2)	−0.0069 (16)	0.0057 (17)	−0.0086 (15)
C9	0.021 (2)	0.025 (2)	0.034 (2)	0.0042 (16)	0.0018 (17)	0.0005 (16)

### Geometric parameters (Å, °)

**Table:**

Te—C1	2.114 (3)	C2—H2A	0.9500
Te—N1	2.421 (3)	C3—C4	1.393 (5)
Te—Cl2	2.446 (8)	C3—H3A	0.9500
Te—Cl3	2.450 (11)	C4—C5	1.381 (5)
Te—Cl1	2.601 (9)	C4—H4A	0.9500
Te—Br3	2.6027 (15)	C5—C6	1.400 (4)
Te—Br2	2.6454 (15)	C5—H5A	0.9500
Te—Br1	2.6945 (17)	C6—C7	1.511 (5)
Te—Cl1^i^	3.414 (9)	C7—H7A	0.9900
Te—Br1^i^	3.5441 (17)	C7—H7B	0.9900
N1—C8	1.473 (4)	C8—H8A	0.9800
N1—C9	1.476 (4)	C8—H8B	0.9800
N1—C7	1.488 (4)	C8—H8C	0.9800
C1—C6	1.387 (4)	C9—H9A	0.9800
C1—C2	1.391 (4)	C9—H9B	0.9800
C2—C3	1.382 (5)	C9—H9C	0.9800
			
C1—Te—N1	76.09 (11)	C8—N1—C7	110.7 (3)
C1—Te—Cl2	88.0 (2)	C9—N1—C7	110.5 (3)
N1—Te—Cl2	84.9 (2)	C8—N1—Te	110.8 (2)
C1—Te—Cl3	92.8 (3)	C9—N1—Te	111.0 (2)
N1—Te—Cl3	167.3 (2)	C7—N1—Te	103.81 (18)
Cl2—Te—Cl3	88.6 (3)	C6—C1—C2	121.7 (3)
C1—Te—Cl1	88.5 (2)	C6—C1—Te	115.9 (2)
N1—Te—Cl1	92.0 (2)	C2—C1—Te	122.3 (2)
Cl2—Te—Cl1	175.8 (3)	C3—C2—C1	119.2 (3)
Cl3—Te—Cl1	93.9 (3)	C3—C2—H2A	120.4
C1—Te—Br3	95.40 (9)	C1—C2—H2A	120.4
N1—Te—Br3	170.80 (7)	C2—C3—C4	119.7 (3)
Cl2—Te—Br3	91.4 (2)	C2—C3—H3A	120.2
Cl1—Te—Br3	91.2 (2)	C4—C3—H3A	120.2
C1—Te—Br2	87.99 (10)	C5—C4—C3	120.9 (3)
N1—Te—Br2	86.50 (8)	C5—C4—H4A	119.5
Cl3—Te—Br2	86.9 (3)	C3—C4—H4A	119.5
Cl1—Te—Br2	176.5 (2)	C4—C5—C6	119.9 (3)
Br3—Te—Br2	89.75 (6)	C4—C5—H5A	120.0
C1—Te—Br1	86.11 (9)	C6—C5—H5A	120.0
N1—Te—Br1	94.91 (8)	C1—C6—C5	118.5 (3)
Cl2—Te—Br1	173.9 (2)	C1—C6—C7	120.2 (3)
Cl3—Te—Br1	90.4 (3)	C5—C6—C7	121.2 (3)
Br3—Te—Br1	87.89 (5)	N1—C7—C6	109.1 (3)
Br2—Te—Br1	173.41 (5)	N1—C7—H7A	109.9
C1—Te—Cl1^i^	171.06 (19)	C6—C7—H7A	109.9
N1—Te—Cl1^i^	97.34 (17)	N1—C7—H7B	109.9
Cl2—Te—Cl1^i^	97.6 (3)	C6—C7—H7B	109.9
Cl3—Te—Cl1^i^	94.3 (3)	H7A—C7—H7B	108.3
Cl1—Te—Cl1^i^	85.6 (3)	N1—C8—H8A	109.5
Br3—Te—Cl1^i^	91.49 (17)	N1—C8—H8B	109.5
Br2—Te—Cl1^i^	97.76 (16)	H8A—C8—H8B	109.5
Br1—Te—Cl1^i^	88.46 (16)	N1—C8—H8C	109.5
C1—Te—Br1^i^	169.81 (9)	H8A—C8—H8C	109.5
N1—Te—Br1^i^	94.83 (7)	H8B—C8—H8C	109.5
Cl2—Te—Br1^i^	95.9 (2)	N1—C9—H9A	109.5
Cl3—Te—Br1^i^	96.7 (2)	N1—C9—H9B	109.5
Cl1—Te—Br1^i^	87.2 (2)	H9A—C9—H9B	109.5
Br3—Te—Br1^i^	93.93 (5)	N1—C9—H9C	109.5
Br2—Te—Br1^i^	96.16 (5)	H9A—C9—H9C	109.5
Br1—Te—Br1^i^	90.15 (4)	H9B—C9—H9C	109.5
C8—N1—C9	109.9 (3)		
			
C1—Te—N1—C8	149.1 (2)	Br3—Te—C1—C6	161.9 (2)
Cl2—Te—N1—C8	59.9 (3)	Br2—Te—C1—C6	72.3 (2)
Cl3—Te—N1—C8	119.3 (12)	Br1—Te—C1—C6	−110.6 (2)
Cl1—Te—N1—C8	−123.0 (3)	Br1^i^—Te—C1—C6	−41.9 (7)
Br2—Te—N1—C8	60.3 (2)	N1—Te—C1—C2	162.6 (3)
Br1—Te—N1—C8	−126.2 (2)	Cl2—Te—C1—C2	−112.2 (4)
Cl1^i^—Te—N1—C8	−37.1 (3)	Cl3—Te—C1—C2	−23.7 (4)
Br1^i^—Te—N1—C8	−35.6 (2)	Cl1—Te—C1—C2	70.1 (3)
C1—Te—N1—C9	−88.6 (2)	Br3—Te—C1—C2	−21.0 (3)
Cl2—Te—N1—C9	−177.7 (3)	Br2—Te—C1—C2	−110.5 (3)
Cl3—Te—N1—C9	−118.4 (12)	Br1—Te—C1—C2	66.5 (3)
Cl1—Te—N1—C9	−0.6 (3)	Br1^i^—Te—C1—C2	135.2 (4)
Br2—Te—N1—C9	−177.4 (2)	C6—C1—C2—C3	−1.9 (5)
Br1—Te—N1—C9	−3.8 (2)	Te—C1—C2—C3	−178.8 (2)
Cl1^i^—Te—N1—C9	85.2 (3)	C1—C2—C3—C4	1.2 (5)
Br1^i^—Te—N1—C9	86.7 (2)	C2—C3—C4—C5	0.0 (5)
C1—Te—N1—C7	30.2 (2)	C3—C4—C5—C6	−0.6 (5)
Cl2—Te—N1—C7	−59.0 (3)	C2—C1—C6—C5	1.3 (5)
Cl3—Te—N1—C7	0.4 (13)	Te—C1—C6—C5	178.4 (2)
Cl1—Te—N1—C7	118.2 (3)	C2—C1—C6—C7	178.4 (3)
Br2—Te—N1—C7	−58.60 (19)	Te—C1—C6—C7	−4.5 (4)
Br1—Te—N1—C7	114.94 (19)	C4—C5—C6—C1	0.0 (5)
Cl1^i^—Te—N1—C7	−156.0 (2)	C4—C5—C6—C7	−177.1 (3)
Br1^i^—Te—N1—C7	−154.50 (19)	C8—N1—C7—C6	−158.5 (3)
N1—Te—C1—C6	−14.5 (2)	C9—N1—C7—C6	79.5 (3)
Cl2—Te—C1—C6	70.7 (3)	Te—N1—C7—C6	−39.6 (3)
Cl3—Te—C1—C6	159.2 (4)	C1—C6—C7—N1	33.4 (4)
Cl1—Te—C1—C6	−107.0 (3)	C5—C6—C7—N1	−149.6 (3)

Symmetry codes: (i) −*x*+1, −*y*+1, −*z*+1.

### Hydrogen-bond geometry (Å, °)

**Table:**

*D*—H···*A*	*D*—H	H···*A*	*D*···*A*	*D*—H···*A*
C7—H7A···Br2^ii^	0.99	2.96	3.839 (4)	149.

Symmetry codes: (ii) *x*−1, *y*, *z*.

## References

[bb1] Addison, A. W., Rao, T. N., Reedijk, J., van Rijn, J. & Verschoor, G. C. (1984). *J. Chem. Soc. Dalton Trans.* pp. 1349–1356.

[bb2] Clark, R. C. & Reid, J. S. (1995). *Acta Cryst.* A**51**, 887–897.

[bb3] Cobbledick, R. E., Einstein, F. W. B., McWhinnie, W. R. & Musa, F. H. (1979). *J. Chem. Res. (S)*, p. 145.

[bb4] Kaur, R., Singh, H. B. & Butcher, R. J. (1995). *Organometallics*, **14**, 4755–4763.

[bb5] Oxford Diffraction (2007). *CrysAlis PRO*, *CrysAlis RED* and *CrysAlis CCD* Oxford Diffraction Ltd, Abingdon, England.

[bb6] Panda, A., Mugesh, G., Singh, H. B. & Butcher, R. J. (1999). *Organometallics*, **18**, 1986–1993.

[bb7] Rivkin, B. B., Maksimenko, A. A. & Sadekov, I. D. (1991). *Zh. Obshch. Khim.* **61**, 1154–1162.

[bb8] Sheldrick, G. M. (2008). *Acta Cryst.* A**64**, 112–122. 10.1107/S0108767307043930 18156677

[bb9] Singh, H. B. & McWhinnie, W. R. (1985). *J. Chem. Soc. Dalton Trans.* pp. 821–825.

[bb10] Singh, H. B., Sudha, N. & Butcher, R. J. (1992). *Inorg. Chem.* **31**, 1431–1435.

[bb11] Singh, H. B., Sudha, N., West, A. A. & Hamor, T. A. (1990). *J. Chem. Soc. Dalton Trans.* pp. 907–913.

